# Research priorities for progressive pulmonary fibrosis in the UK

**DOI:** 10.1136/bmjresp-2024-002368

**Published:** 2024-09-03

**Authors:** Laura Fabbri, Anne-Marie Russell, Nazia Chaudhuri, Wendy Adams, Katherine Cowan, John Conway, Wendy Dickinson, Michael Gibbons, Simon Hart, Steve Jones, Jenny Lynch-Wilson, Tom McMillan, Steve Milward, Maureen Ward, Louise Elisabeth Wright, Gisli Jenkins

**Affiliations:** 1 National Heart and Lung Institute, Imperial College London Department of Medicine, London, UK; 2 Department of Respiratory Medicine, Royal Brompton Hospital, London, UK; 3 Nottingham Biomedical Research Centre, National Institute for Health Research, Nottingham, UK; 4 College of Medical and Dental Sciences (MDS) University of Birmingham, Birmingham, UK; 5 University of Exeter, Exeter, UK; 6 University of Ulster Faculty of Life and Health Sciences, Londonderry, UK; 7 Action for Pulmonary Fibrosis, Peterborough, UK; 8 James Lind Alliance, Southampton, UK; 9 Katherine Cowan Consulting Limited, East Sussex, UK; 10 Patient Representative, Tooting, UK; 11 Carer Representative, Nottingham, UK; 12 Respiratory Medicine, Royal Devon & Exeter NHS Foundation Trust, Exeter, UK; 13 Respiratory Research Group, Hull York Medical School/University of Hull, Cottingham, UK; 14 Hywel Dda University Health Board, Carmarthen, UK; 15 Carer Representative, Ballycastle, UK; 16 Patient Representative, Bolton, UK; 17 Carer Representative, Tameside, UK

**Keywords:** interstitial fibrosis, idiopathic pulmonary fibrosis

## Abstract

**Introduction:**

Health research bodies recommend patient involvement and engagement in research and healthcare planning, although their implementation is not yet widespread. This deficiency extends to progressive pulmonary fibrosis (PPF), where crucial aspects remain unknown, including causal mechanisms, curative treatments and optimal symptom management. This study addresses these gaps by seeking stakeholders’ perspectives to guide research and treatment directions.

**Method:**

A priority-setting partnership was established to explore stakeholders’ priorities in the diagnosis, treatment, management and care of PPF, including idiopathic pulmonary fibrosis which is the archetypal PPF. Stakeholders included people living with PPF, their carers, relatives and healthcare professionals involved in their management.

**Results:**

Through an online open-ended survey, 2542 responses were collected from 638 stakeholders. Thematic analysis identified 48 specific research questions, which were then cross-referenced with academic literature to pinpoint research gaps. Following the evidence check, 44 unanswered questions were shortlisted by 834 stakeholders in a second online survey. Ultimately, a top 10 priority list was established through consensus.

The prioritised research questions include (1) improved diagnosis accuracy and timing, (2) development of new treatments, (3) enhanced accuracy in primary care, (4) optimal timing for drug and non-drug interventions, (5) effective cough treatment, (6) early intervention for PPF, (7) improved survival rates, (8) symptom reduction, (9) impact of interventions on life expectancy and (10) new treatments with reduced side effects.

**Conclusion:**

Stakeholders’ priorities can be summarised into five areas: early diagnosis, drug and non-drug treatments, survival and symptom management. Ideally, these topics should guide funding bodies and health policies.

**WHAT IS ALREADY KNOWN ON THIS TOPIC**

- Health research bodies recommend the involvement and engagement of stakeholders in research and healthcare planning, but this was not applied to progressive pulmonary fibrosis (PPF), where crucial aspects remain unknown.

**WHAT THIS STUDY ADDS**

- Stakeholders consider early diagnosis, drug and non-drug treatments, survival and symptom management key priority areas for future research in PPF.

**HOW THIS STUDY MIGHT AFFECT RESEARCH, PRACTICE OR POLICY**

- The highlighted topics should guide the decisions of funding bodies and health policies.

## Introduction

 Integration of patient and public involvement and engagement (PPIE) was formally identified as an urgent priority about 20 years ago, particularly in the UK, where it represents a founding principle of the National Institute of Health Research (NIHR).^1^ Notwithstanding the acknowledged importance of public involvement, its implementation in everyday research has been slow.^2^ We know that PPIE can improve healthcare quality, health literacy and patient autonomy, with positive effects on patients’ health and cognitive and emotional empowerment, and it is cost-effective.^3 4^

Interstitial lung diseases (ILDs) are a group of fibroinflammatory diseases that lead to inflammation or scar tissue deposition (fibrosis) within the alveolar interstitium. ILDs can be of unknown cause or be associated with other diseases or environmental exposures.^5^ They have an incidence of between 1 and 31.5 per 100 000 person-years and a prevalence between 6.3 and 71 per 100 000 people. Some patients can have a chronic and relentless evolution, and the term *progressive pulmonary fibrosis* (PPF) is used to describe the cases of pulmonary fibrosis (PF) that have a fibrotic phenotype. While recent PPF guidelines refer to ILDs other than idiopathic pulmonary fibrosis (IPF),^6 7^ for the purpose of this study, IPF has been included in the definition of PPF as IPF is the archetypal progressive ILD. PPF is associated with worsening respiratory symptoms, a decline in lung function, decreased quality of life and a risk of early death.^8^ Many aspects of PPF remain unknown, such as the causal mechanisms, a defined curative treatment or best symptom management. For its severity and poor prognosis, PPF is often compared with cancer. Yet, for years, stakeholders across Europe have reported a lack of services and difficulties in getting a diagnosis, treatment access, holistic care and palliative care,^9 10^ care that is more widely available to patients with cancer.^11 12^

This study aimed to identify stakeholders’ views on PPF and to prioritise these unknowns to inform policymakers, funders, researchers, industry and others who have the potential to implement the priorities identified.

## Methods

### The priority setting partnership

**Figure 1:**
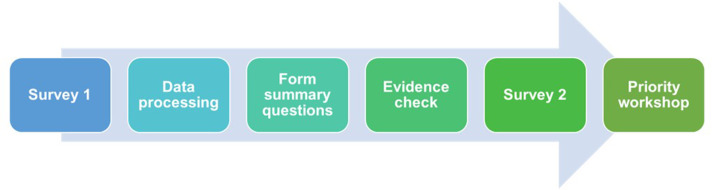
Schematic representation of James Lind Alliance methodology.

An established methodology, designed by the James Lind Alliance (JLA), was used. This methodology was developed to bring patients, carers and clinicians together in a priority setting partnership (PSP) to identify uncertainties or unanswered questions about specific health issues. A PSP is a multistep process: data are collected through a first survey and then successively processed to formulate research questions. After a check of the literature, the research questions that still have to be investigated will go through a second survey to be shortlisted. Finally, a ‘top 10’ list of those uncertainties for research is agreed by consensus during a workshop (figure 1).^13^

The steering group for our PSP included people diagnosed with PF (n=4), carers (n=3) and healthcare professionals (HCPs) (n=6) with expertise in PPF, who met online 11 times over 2 years. This group led the PSP under the guidance of a JLA’s senior advisor. Steering group members were purposively invited from the charity (Action for Pulmonary Fibrosis) network. The sampling followed a strict balance of group representation (patients, carers, HCPs), geographic and gender distribution. The steering group agreed to limit the scope of the PSP to diagnosis, treatment, management and care of PF. The first survey was limited to the UK, while the second was opened worldwide, although most entries (94%) were received from the UK.

### The first survey

Initially, we gathered stakeholders’ questions and uncertainties through an online survey with open-ended questions. The questions, developed with patient partners, reflected the in-scope themes: (a) *What questions or concerns about the diagnosis of PF would you like to see answered by research?* (b)*What questions or concerns about the treatment of PF would you like to see answered by research?* (c)*What questions or concerns about the care of people affected by PF would you like to see answered by research?* Stakeholders included people living with PF, carers, relatives and HCPs involved in their management. A secure Research Electronic Data Capture cloud was used for data collection.

The survey was advertised on social media (Facebook, LinkedIn, Twitter, Instagram), in newsletters, through the patient group networks and support partners and through word of mouth. To ensure the participation of those unfamiliar with technology, paper copies of the survey were mailed to members of patient support groups. Participants had the option to register their interest to receive feedback on the outcomes of the study.

After the closure of the survey, data were anonymised, and a thematic inductive approach was used. LF identified ‘keywords’ themes which were coded generating a codebook. LF and WA identified patterns in the coded data and grouped similar codes to generate overarching themes. The themes were mapped against the preset criteria in and out of scope. From each in-scope theme, a summary research question was generated using consensus agreement from the wider team. In case of disagreement, data were discussed with the independent advisor KC, and decided by three-way consensus. We acknowledge that theme generation can be affected by the perceptions and experiences of individuals analysing data (LF and WA) and include a statement of reflexivity (online supplemental file 1).

The summary questions were worded in lay language, avoiding jargon, to be understood by lay audiences. The steering group reviewed the list of research questions to determine whether they adequately reflected the survey submissions. Three separate working groups were organised for a first round of reviews, and results were successively discussed collegially and agreed on by steering group consensus. The independent advisor facilitated all meetings in line with the JLA methodology.^13^ A targeted literature review was performed to verify the research questions identified in the online survey. We chose a pragmatic approach as a full systematic review was impractical within the timeframe of the PSP. Targeted review criteria were as follows: (a) to ensure retrieval of the most recent literature, searches were restricted from 2015 to 2021, (b) search was limited to English-only articles, studied in humans and restricted to adults, (c) guidelines, systematic reviews, review, articles and articles in-press were included, (d) abstracts, editorials and research letters were excluded. We consulted the databases EMBASE, MEDLINE, CINAHL and Google Scholar. Given the broad scope of our PSP, the research questions that were derived by the above methodology were considered unanswered if there was no systematic review, a recent systematic review indicated insufficient evidence or insufficient evidence outlined in consensus papers. Literature search results were submitted to the JLA Institute for transparency. Results were rediscussed within the steering group. Research questions inadequately covered in the literature were selected for the second survey.

### The second survey and the workshop

For the second survey, we asked stakeholders to select up to 10 questions which they considered most important from the 44 derived from the first survey. We used the webpage SurveyMonkey for the poll.^14^ The 15 out of 44 top-rated questions selected were subsequently ranked during an online workshop.

Workshop participants were purposively sampled to ensure a balanced representation of people living with PF, carers and HCPs with adequate expertise and representation from all the nations and regions in the UK, as well as gender. The consultation was split over two 3-hour sessions run on consecutive days. Participants were initially divided into four equally representative groups, each supported by a JLA moderator. Each group debated the importance of all questions, evaluating urgency, pros and cons and ranked all 15 questions in order of priority. Results were subsequently merged and rediscussed during a plenary session the following day. The final top 10 ranking was agreed by consensus.

Reporting follows the guideline for priority setting of health research (REPRISE) (online supplemental file 2).^15^

### Patient and public involvement

Patient and public involvement was the driving force behind this project, which was co-produced by the patient-led charity Action for Pulmonary Fibrosis. Charity representatives had a significant role in designing, recruiting participants and disseminating results. The steering group included people living with the disease and carers, who are also coauthors of this paper.

## Results

### First survey results

The initial online survey was open from April to May 2021. 638 UK participants completed the survey and generated 2542 single statements/questions. Details about the demographic data are presented in table 1. HCPs worked in primary (29%), secondary (40%) and tertiary care (21%), and 43% of them had >10 years of experience working with ILDs. Geographic distribution was 81% in England, 6% in Scotland and 5% in Wales and Northern Ireland, respectively.

**Table 1:** Demographics (A) participants; (B) self-reported data on underlying ILD; (C) HCPs categories

(A) Participants						
Participants	First survey		Second survey		Workshop	
	N	%	N	%	N	%
Person living with PF	362	57	429	51	7	32
Carer/Relative	163	25	273	33	4	18
Healthcare professional	93	15	132	16	8	36
Other*	20	3	NA	NA	3	14
Total	638	100	834	100	22	100

(B) Self-reported data on underlying ILD				
People living with PF, carers and relatives	First survey		Second survey	
	N	%	N	%
IPF	361	69	486	62
FPF	12	2	25	3
NSIP	21	4	30	4
cHP	24	5	22	3
uILD	10	2	13	1
CTD-ILD	28	6	53	8
Occupational ILD	12	2	4	1
Sarcoidosis	20	5	9	1
Other†/Blank	37	5	59	13

(C) HCPs categories				
HCPs	N	%	N	%
Physician	31	33	53	40
Physiotherapist	12	13	14	11
Pharmacist	2	2	1	1
Palliative care	1	1	3	2
Nurse	33	35	44	33
General practitioner	9	10	5	4
Dietitian	1	1	0	0
Psychologist	0	0	3	2
Other†/Blank	4	4	9	7

* Participants could classify themselves as other if thinking to fit in more than one category.

† Participants could classify themselves as other if thinking to fit in more than one category, or leave the box blank.

cHP, chronic hypersensitivity pneumonitis; CTD-ILD, connective tissue disease-related ILDFPF, familial pulmonary fibrosis; ILD, interstitial lung disease; IPF, idiopathic pulmonary fibrosis; NA, not applicable; NSIP, non-specific interstitial pneumonia; PFpulmonary fibrosisuILD, unclassifiable ILD

The in-scope entries were distilled into 48 themes and subsequent research questions. After review and collegial discussion by the steering group, 44 questions (box 1) were shortlisted for the second survey.

Box 1 **Unresolved research questions**

- What tests and tools (eg, blood tests, lung function, imaging, virtual and artificial technology) can predict the progression of progressive pulmonary fibrosis (PPF)?
- How many people live with different types of PPF in the UK?
- How can acute deteriorations of PPF be predicted in patients with PPF?
- Can new treatments for PPF be developed with reduced side effects? Does how the drug is delivered (eg, oral, nebulised, through a vein) affect potential side effects of the drug in PPF?
- Can treatments halt or reverse PPF?
- What are the increased medical risks following a diagnosis of PPF during certain medical procedures (eg, anaesthesia), and how can these be reduced or eliminated?
- How should exercise programmes and pulmonary rehabilitation be delivered to best improve symptoms and quality of life in PPF?
- How can the delivery of portable and home-based oxygen be improved (digital monitors, remote control, lighter weight, quieter, higher flow rates) for patients with PPF?
- What is the best time to refer to occupational therapy to benefit quality of life and improve planning for the future for patients with PPF and their carers?
- What are the biological changes in human cells that lead to the development of PPF?
- What can be done to improve the speed and accuracy of PPF diagnosis in primary care (eg, training, integration of case-based studies in general practice training, awareness campaigns)? 
- How can we use new technology (eg, artificial intelligence) to help inform diagnosis and prognosis of PPF?
- What is the best time for drug and non-drug interventions (pulmonary rehabilitation, oxygen therapy, psychological support) to start to preserve quality and length of life for patients with PPF?
- To what extent do different interventions (medication, pulmonary rehabilitation, oxygen therapy, psychological support) impact length of life in patients with PPF? 
- What is the best management of acute deterioration in PPF?
- How can treatments be tailored for individual patients with PPF?
- What forms of education and training for healthcare professionals could improve the way patients and families are informed of the diagnosis of PPF?
- Can non-drug interventions (eg, yoga, singing, relaxation techniques, acupuncture, herbal remedies, etc) improve well-being, symptoms management and survival in PPF?
- Does diet help with the management of PPF symptoms?
- What treatments (drug, non-drug and aids) can reduce breathlessness and phlegm production in PPF?
- Does psychological well-being affect PPF disease progression?
- What type of support (psychological, peer, drug) is most effective at reducing feelings of isolation, depression and anxiety in patients, carers and families affected by PPF?
- Are there health inequalities in access to care for PPF (eg, ethnic minorities or gender differences)? If so how can these be reduced?
- What tests and tools (eg, blood tests, lung function, imaging, virtual and artificial technology) should be used to monitor progression of PPF?
- What are the most effective ways to reduce or manage side effects from medications used to treat PPF?
- How can other co-existing medical conditions (comorbidities) be managed in people living with PPF?
- How can the diagnosis of PPF be improved in terms of accuracy and the time taken (screening programme, early signs and symptoms that could be detected in primary care, blood markers, imaging, biopsy, artificial intelligence, etc)?
- What is the optimum timing for lung transplantation in PPF?
- What treatments (drug, non-drug and aids) can treat cough in PPF?
- Would early treatment delay progression, lung function decline and improve survival in PPF?
- Which therapies will improve survival in PPF?
- How can palliative care support be more acceptable for people living with PPF, and when should this be proposed? 
- Can the likelihood of developing PPF be predicted through genetic screening?
- How does geography impact on the quality of care that a person with PPF receives?
- What is the most effective multidisciplinary team structure and function to support patients and families affected by PPF?
- What are the psychological consequences of a diagnosis of PPF for patients, their families and carers?
- Can oxygen improve quality of life and outcomes in PPF?
- How can the discussion and management of end of life in PPF be improved so that patients and families feel better prepared and supported?
- How can peer support (support groups, befrienders, friends) impact disease management for patients with PPF and their carers?
- What support (eg, information and training, financial, psychological, etc) would enable carers of patients with PPF to feel empowered in their role?
- To what extent can different interventions (medication, pulmonary rehabilitation, oxygen therapy, psychological support) impact quality of life in patients with PPF? 
- How effective are different treatments at treating different types of PPF?
- Can drugs used to treat other diseases be effective in treating PPF?
- Can treatments other than pirfenidone and nintedanib slow the progression of PPF?

Initial 44 questions identified by the first survey, after the targeted literature review.Note, the order is random, no priorities.

### Second survey results

The second survey was online in April and May 2022. 834 stakeholders took part in this survey and submitted their selections. Details about the demographic data are reported in table 1. In this survey, HCPs were mainly from tertiary (45%) and secondary (40%) care, and 49% of them had >10 years of experience working with ILDs. Geographical distribution was 77.7% in England, 8.2% in Wales, 6.2% in Scotland, 4.6% in Northern Ireland and 3.2% outside the UK.

Notably, the top 15 ranking questions shortlisted during this survey were equivalent across people living with the disease, carers and HCPs (table 2).

**Table 2:** Interim rankings for the top 15 research questions (top questions for each group from the long list of 44)

Rank	Question	Patients	Carers	HCPs
A	Can new treatments for PPF be developed with reduced side effects? Does how the drug is delivered (eg, oral, nebulised, through a vein) affect potential side effects of the drug in PPF?	9	12 =	13 =
B	Can new treatments other than pirfenidone and nintedanib slow, halt or reverse the progression of PPF?	1	4	2 =
C	Can the likelihood of developing PPF be predicted through genetic screening?	23 =	1 =	7
D	How can acute deteriorations of PPF be predicted in patients with PPF?	16	16 =	9
E	How can the delivery of portable and home-based oxygen be improved (digital monitors, remote control, lighter weight, quieter, higher flow rates) for patients with PPF?	10	9	16 =
F	How can the diagnosis of PPF be improved in terms of accuracy and the time taken (screening programme, early signs and symptoms that could be detected in primary care, blood markers, imaging, biopsy, artificial intelligence, etc)?	12 =	5	6
G	To what extent do different interventions (pulmonary rehab, oxygen therapy, psychological support) impact length of life in patients with PPF?	11	20	26
H	What are the biological changes in human cells that lead to the development of PPF?	27	10 =	11
I	What can be done to improve the speed and accuracy of PPF diagnosis in primary care (eg, training, integration of case-based studies in general practice training, awareness campaigns)?	7	3	18 =
J	What is the best management of acute deterioration in PPF?	17	15	2 =
K	What is the best time for drug and non-drug interventions (pulmonary rehab, oxygen therapy, psychological support) to start to preserve quality and length of life for patients with PPF?	8	16 =	10
L	What treatments (drug, non-drug and aids) can reduce breathlessness and phlegm production in PPF?	2	8	20
M	What treatments (drug, non-drug and aids) can treat cough in PPF?	6	10 =	1
N	Which therapies will improve survival in PPF?	5	6 =	8
O	Would early treatment delay progression, lung function decline and improve survival in PPF?	3	1 =	2 =

‘=’ denotes joint place.

PPFprogressive pulmonary fibrosis

### Workshop

In June 2022, 15 out of the 44 most voted-for questions were discussed during an online workshop, where the priorities were ranked to define a top 10 (table 3). This was done by 22 stakeholders—7 living with PF, 4 carers, 8 HCPs and 3 who fitted more than one category (eg, carer and HCP). All of them were recruited in the UK.

**Table 3:** Top 10 research priorities for progressive pulmonary fibrosis (PPF)

Rank	Question
1	How can the diagnosis of PPF be improved in terms of accuracy and the time taken (screening programme, early signs and symptoms that could be detected in primary care, blood markers, imaging, biopsy, artificial intelligence, etc)?
2	Can new treatments other than pirfenidone and nintedanib slow, halt or reverse the progression of PPF?
3	What can be done to improve the speed and accuracy of PPF diagnosis in primary care (eg, training, integration of case-based studies in general practice training, awareness campaigns)?
4	What is the best time for drug and non-drug interventions (pulmonary rehab, oxygen therapy, psychological support) to start to preserve quality and length of life for patients with PPF?
5	What are the best ways (drug, non-drug and aids) to treat cough in PPF?
6	Would early treatment delay progression, lung function decline and improve survival in PPF?
7	Which therapies will improve survival in PPF?
8	What treatments (drug, non-drug and aids) can reduce breathlessness and phlegm production in PPF?
9	To what extent do different interventions (pulmonary rehab, oxygen therapy, psychological support) impact length of life in patients with PPF?
10	Can new treatments for PPF be developed with reduced side effects? Does how the drug is delivered (eg, oral, nebulised, through a vein) affect potential side effects of the drug in PPF?

Within the top 10 selections, we identified 5 main themes.

*Diagnosis (questions 1 and 3)*: stakeholders acknowledged the challenges of getting an early diagnosis. Early detection is desirable and may occur through screening programmes, or tests with high sensitivity and specificity. An important point highlighted is to prioritise the education of primary care HCPs to recognise early signs and red flags for a swift referral to specialist centres. Stakeholders highlighted misdiagnosis causing delays in referral to specialist services, as initial symptoms such as dyspnoea and cough are not specific to PF.

*Drug treatments (questions 2, 6and 10)*: new drug development was a pivotal priority, particularly addressing the need for a cure. Research should strive to stop and reverse the fibrotic process. At the time of the survey, in the UK, antifibrotic prescribing for IPF was restricted to those with a forced vital capacity (FVC) between 50% and 80% of predicted values. It was therefore deemed critical by stakeholders to explore the need for more data about the optimal timing to start treatment. Stakeholders wanted to know the correct timing to start treatment to prevent the progression of the disease, reduce the decline in lung functions and improve the survival rate. The uncertainties about timing were also impacted by concerns related to the side effects of treatment and the impact on the patient’s quality of life. Stakeholders identified the need for new treatments or new administration routes with reduced side effects.

*Non-pharmacological treatments (questions4 and 9)*: stakeholders highlighted concerns regarding the side effects of drugs and their impact on quality of life as a reason for significant interest in non-pharmacological approaches to management, including pulmonary rehabilitation, oxygen therapy, psychological support and peer support. It was acknowledged that there may be insufficient data on the efficacy and best timing for these approaches, particularly pertinent to people who could not tolerate drug therapy or would like to explore other alternative types of therapy.

*Survival (question 7)*: poor prognosis was highlighted by stakeholders as a key distressing point, with ‘3–5 years survival’ as a recurrent statistic used in healthcare conversations. Therefore, identifying novel therapies to improve survival was deemed a valid research point on its own, irrespective of current pharmacological and non-pharmacological treatments.

*Symptom management (questions5 and 8)*: symptoms caused by PF can be disabling. Productive cough and dyspnoea impact the quality of life and are perceived as a cause of stigma. Stakeholders are interested in any possible treatment, including drug, non-drug or aids that could reduce these symptoms or help manage them.

The research question about the causes of PF, worded as ‘What are the biological changes?’ was not included in the top 10. Other excluded themes discussed during the workshop that were considered necessary but did not reach the priority of the top 10 are the prediction and management of acute exacerbation, the efficacy of genetic screening and how to improve oxygen devices (table 2).

## Discussion

Qualitative work has explored broad concepts related to clinical care pathways, but it is often limited to small groups^15 16^ or to countries outside the UK.^16^ We acknowledge that patient populations worldwide have similar needs. Still, there are different nuances due to the structure of health service provision and deficits in many areas of health service provision.^17^

The design of this project was developed at a time when there was ongoing discussion about grouping ILD based on their phenotypes. In the subsequent years, experts deliberated on this issue, which eventually led to the publication of documents by ATS and ERS, although with some controversy.^18^ It is important to mention that we included both IPF and non-IPF fibrotic ILD in our definition of PPF.

Our primary strength is the coproduction with the patient-led groups, allowing us to distribute the survey capillary with broad coverage. To ensure that participants who were less familiar with technology could be included, we were not limited to an online survey and included paper copies. The JLA offers a robust methodology focused on research priorities, ensuring that future research is patient-centred. From our study, the first in the UK with a broad cohort and following the JLA methodology, stakeholders consider that early diagnosis, new drug and non-drug treatments, survival and symptom management require prioritisation.

Early diagnosis was the highest ranked priority. Delays in the diagnostic pathway are a well-recognised problem worldwide.^19^,^21^ The time from the onset of symptoms to diagnosis can reach up to 24 months.^19^ Delayed diagnosis has been proven to negatively impact progression-free survival, quality of life and hospitalisation rates in patients with IPF.^19^ Delays may happen in several stages of the diagnostic pathway, due to under-reporting of ILD features on diagnostic testing and prolonged time to pulmonology referral even when ILD is reported.^22^ As reported by a similar study in Australia,^16^ there is an educational need for training for primary care HCPs and also a research interest in new technologies, such as biomarkers, artificial intelligence and imaging, which can be used to detect ILD earlier.^23 24^

After diagnosis, finding a treatment to cure fibrosis is a priority. Pirfenidone and nintedanib represented a paradigm shift in managing IPF and progressive fibrotic ILD, but stakeholders require fibrosis to be stopped and reversed—slowing it down is not enough. Future research might consider targeting distinct pathogenetic pathways or focusing on alveolar regenerative medicine.^25^ Other approaches to consider are combining treatments or subcategorising patients according to comorbidities and biomarkers for tailored care.^26^

The best timing of antifibrotic treatment was deemed a priority specifically related to the NICE requirements about the FVC threshold for an antifibrotic prescription, which applied when we ran the survey. At the time of the survey, FVC had to be between 50% and 80% of the predicted value to be eligible for antifibrotics prescription. To date, in the UK, these limits still apply to pirfenidone, while for nintedanib the 80% threshold has been removed for patients with IPF and its licence has been extended to progressive fibrotic ILD.^27 28^ Antifibrotics slow the loss of lung function, and there is evidence that early treatment may also improve survival. However, these data require further investigation, as they may be misinterpreted due to lead-time bias: earlier detection may only move forward the time of a patient’s treatment start without moving back the time of death.^26 29 30^

Furthermore, we should not overlook the downside of antifibrotics, which emerged in the priorities listed. Side effects, particularly gastrointestinal ones, can affect up to 30% of patients and impact their quality of life.^31^ Although patients try to cope with them, they are the main reason why patients stop pharmacological treatment.^32 33^ Reducing and managing side effects is one of the priorities listed.

Besides the pharmacological approach, our stakeholder group also prioritised non-drug management scenarios. The benefits of a holistic approach, including pulmonary rehabilitation, oxygen supplementation, palliative interventions, lung transplant, psychological support and peer support, have not been sufficiently proven and quantified.^34^ There is some evidence for pulmonary rehabilitation and oxygen,^35^,^37^ but there are still unanswered questions. This applies especially to symptom management, cough and dyspnoea, which are perceived as invalidating and stigmatising.^38^

Some of the priorities listed are similar to those identified by Tikellis *et al*,^16^ particularly those related to the patients’ experience with delayed diagnosis, symptom management, drug side effects and the need for a cure. These similarities suggest that countries with developed healthcare systems have common priorities, and it may not be necessary to repeat local consultation but to dedicate resources for specific service improvements. Differently from the Australian colleagues, we purposefully excluded researchers from the workshop participants to reduce the risk of bias and amplify the voices of stakeholders who do not usually influence the research agenda (as per JLA principles). This may be the reason why stakeholders deprioritised the question about the importance of basic science and translational medicine, not apparent to the general public. This finding should prompt the development of public engagement activities in basic science. Similarly, acute exacerbations were deemed of interest only by HCPs, who are used to managing them, while for patients and carers, acute exacerbations represent only the inevitable end point. Likewise, oxygen devices were considered of interest only for patients with more severe diseases and, therefore, deprioritised during the discussion. An interesting and polarising point of discussion was genetic screenings. Some participants wished this was widely available to test their children, while others had strong positions against it in the context of no cure available. However, a previous study showed that relatives of patients with PF who undergo screening for early disease do not regret the experience.^39^

We acknowledge the limits of our investigation. For pragmatic reasons, we predefined the topics to be considered in scope. Despite our best efforts to involve a diverse and representative population, linguistic barriers may have prevented some people from taking part, as surveys and workshops were available only in English. Also, since the participants were all volunteers, there may have been a self-selection bias, with only highly motivated people participating in the research. We campaigned to enrol people living with different types of PF. Still, IPF represented the majority (69% and 62%) of the survey participants and their opinions may have dominated the inputs compared with non-IPF participants. Given the nature of the data (participants inputted their own data; no medical records were consulted), we cannot comment on the severity or progression of their condition or exclude that some participants had a more stable disease, which might not be defined as progressive. The top 10 priorities were agreed on through consensus in a workshop, and participants were purposively invited to balance the different stakeholders’ groups (patients, carers and HCPs), geographic distribution and gender. To reduce bias, researchers were excluded from the recruitment and data collection for the workshop, and the charity Action for Pulmonary Fibrosis invited the participants from their network. We cannot exclude that the sampling of stakeholders influenced the priority list, and a different cohort of participants with different experiences and expertise in PPF may have chosen different priorities.

## Conclusion

Our study identified a comprehensive list of topics which have yet to be adequately investigated, including the diagnosis, treatment, management and care of PPF. Stakeholders consider that early diagnosis, new drug and non-drug treatments, survival and symptom management require prioritisation. Our findings should ideally guide funding bodies, health policies and researchers to ensure resources are focused on projects that matter to the people affected by PPF.

## supplementary material

10.1136/bmjresp-2024-002368 online supplemental file 1

10.1136/bmjresp-2024-002368 Uncited online supplemental file 2

## Data Availability

Data are available on reasonable request.
