# Supplementary material for: Research priorities for progressive pulmonary fibrosis in the UK
Source: BMJ Open Respir Res. 2024 Sep 3;11(1):e002368. doi: 10.1136/bmjresp-2024-002368 (PMC11428979; doi:10.1136/bmjresp-2024-002368)
Supplement: online supplemental file 1 [file bmjresp-11-1-s001.pdf]

Supplement 1 - Reflexivity statement

People affected by pulmonary fibrosis face considerable unmet needs from pre-diagnosis to diagnosis, management and care. Research offers an opportunity to overcome some of these areas of need, yet resources are scarce, and prioritisation is necessary. Research priorities are typically set by academia and industry, but their priorities may differ from the priorities of those affected by the disease or healthcare professionals supporting the management of the disease. The James Lind Alliance Priority Setting Partnership process enabled people affected by progressive pulmonary fibrosis and healthcare providers to identity their top 10 priorities for research.

LF and WA entered the process of the JLA with the aim of enabling the voices of people affected by PF and healthcare professionals to be heard. LF and WA had a significant role in generating themes from coded data. Throughout the PSP process, reflexivity for LF and WA involved:

- Maintaining a commitment to transparency: openly sharing their process with the PSP panel including examples of raw data, how these were coded and theme allocation
- Seeking guidance and direction from the PSP panel to guide all aspects of their work
- Aiming to authentically represent the views of the survey respondents, putting aside personal or academic interest in the outcome of the process
- Maintaining an openness to novel ideas and theme generation

Although LF and WA have extensive knowledge of progressive pulmonary fibrosis, they were acutely aware that the data being generated were novel, and the outcomes could and should not be predicted. They approached the data with curiosity and openness, allowing insight from the data, rather than existing knowledge, to generate themes. That said, it is not always possible to be aware of subconscious biases, and the authors acknowledge that naïve coders may have grouped data differently.

LF acknowledges her standpoint as a non-British white, early-career medical doctor and researcher with an interest in ILD and lived experience with chronic progressive disease. This project was also part of her PhD research. As a healthcare professional and a person with lived experience in chronic progressive disease, the apparent dichotomy of her background offered an insider position in data review and theme generation.

WA acknowledges her position as a white British, doctoral educated, patient advocacy organisation staff member. As a patient advocacy representative, her values to compassionately and accurately give visibility to the needs and priorities of the pulmonary fibrosis community were apparent in this process.
