# Supplementary material for: Research priorities for progressive pulmonary fibrosis in the UK
Source: BMJ Open Respir Res. 2024 Sep 3;11(1):e002368. doi: 10.1136/bmjresp-2024-002368 (PMC11428979; doi:10.1136/bmjresp-2024-002368)
Supplement: Uncited online supplemental file 2 [file bmjresp-11-1-s002.pdf]

No	Item	Descriptor and/or examples	Page
A	Context and scope		
1	Define geographical scope	Global, regional, national, city, local area, institutional/organizational level, health service	4
2	Define health area, field, focus	Disease or condition specific, interventions, healthcare delivery, health system	3
3	Define the intended beneficiaries	This may include the general population or a specific population based on demographic (age, gender), clinical (disease, condition), or other characteristics who may benefit from the research	3
4	Define the target audience of the priorities	Policy makers, funders, researchers, industry or others who have the potential to implement the priorities identified	3
5	Identify the research area	Public health, health services research, clinical research, basic science	4
6	Identify the type of research questions	Etiology, diagnosis, prevention, treatment (interventions), prognosis, health services, psychosocial, behavioral and social science, economic evaluation, implementation; this may not be pre-defined	4
7	Define the time frame	Interim, short-term, long-term priorities, plans to revise and update	NA
B	Governance and team		

8	Describe the selection and structure of the leadership and management team	Those responsible for initiating, developing, and guiding the process for priority setting, and examples of structures include; Steering Committee, Advisory Group, Technical Experts	4
9	Describe the characteristics of the team	Stakeholder group or role, institutional affiliations, country or region, demographics (e.g. age sex), discipline, experience, expertise	4
10	Describe any training or experience relevant to conducting priority setting	Consultants or advisors, members with experience or skills relevant to the conducting priority-setting e.g. qualitative methods, surveys, facilitation	NA
C	Framework for priority setting		
11	State the framework used (if any)	James Lind Alliance, COHRED, CHNRI, Dialogue Model, no framework (general research priority setting)	3-4
D	Stakeholders or participants		
12	Define the inclusion criteria for stakeholders involved in priority-setting	Patients, caregivers, general community, health professionals, researchers, policy makers, non-governmental organizations, government, industry; specific groups including vulnerable and marginalized populations	4
13	State the strategy or method for identifying and engaging stakeholders	Partnership with organizations, social media, recruitment through hospitals	4-5

14	Indicate the number of participants and/or organizations involved	Number of individuals and organizations, include number by stakeholder group	6-8
15	Describe the characteristics of stakeholders	Stakeholder group, demographic characteristics, areas of interest and expertise, discipline, affiliations	6-8
16	State if reimbursement for participation was provided	Cash, vouchers, certificates, acknowledgement; what purpose e.g. travel, accommodation, honorarium	NA
E	Identification and collection of research priorities		
17	Describe methods for collecting initial priorities	Methods e.g. Delphi survey, surveys, nominal group technique, interviews, focus groups, meetings, workshops; prioritization e.g. voting, ranking; mode e.g. face-to-face, online; may be informed by evidence e.g. systematic reviews, reviews of guidelines/other documents, health technology assessment	4-5
18	Describe methods for collating and categorizing priorities	Taxonomy or other framework used to organize, summarise, and aggregate topics or questions	4-5
19	Describe methods and reasons for modifying (removing, adding, reframing) priorities	Based on scope, clarity, definition, duplication, other criteria	4-5
20	Describe methods for refining or translating priorities into research topics or questions	Reviewed by Steering Committee or project team	4-5

21	Describe methods for checking whether research questions or topics have been answered	Systematic reviews, evidence mapping, consultation with experts	4-5
22	Describe number of research questions or topics	Number of priorities at each stage of the process	6-8
F	Prioritization of research topics/questions		
23	Describe methods and criteria for prioritizing research topics or questions	Methods e.g. Delphi survey, surveys, nominal group technique, interviews, focus groups, meetings, workshops;	7
24	State the method or threshold for excluding research topics/questions	Thresholds for ranking scores, proportions, votes; other criteria	4-5
G	Output		
25	State the approach to formulating the research priorities	Area, topic, questions, PICO (population, intervention, comparator, outcome)	4-5
H	Evaluation and feedback		
26	Describe how the process of prioritization was evaluated	Survey, workshop	NA
27	Describe how priorities were fed back to stakeholders and/or to the public; and how feedback (if received) was addressed and integrated	Public meetings or workshop, newsletters, website, email, online presentations	NA
I	Implementation		

28	Outline the strategy or action plans for implementing priorities	Communication with target audience, via policies and funding	8-11
29	Describe plans, strategies, or suggestions to evaluate impact	Integration in decision-making, funding allocation, review of relevant documents	NA
J	Funding and conflict of interest		
30	State sources of funding	Name sources of funding for the priority-setting exercise; if relevant include the budget and/or cost	6
31	Declare any conflicts or competing interests	State any conflicts of interest that may be at an individual level and/or at a contextual level (e.g. political issues, controversies) that may affect the process, output or implementation.	20
